# Supplementary material for: Analysis of dendritic input currents during place field dynamics
Source: eLife. 2026 Jul 3;14:RP108352. doi: 10.7554/eLife.108352 (PMC13331480; doi:10.7554/eLife.108352)
Supplement: Peer review history

# Editor report: eLife Assessment

**DOI:** 10.7554/eLife.108352.3.sa0

**Authors:** Christine Grienberger

**Affiliations:** Brandeis University United States

**Keywords:** Convincing, Important

This study offers an **important** advance by extending an intuitive visualization tool that enables assessment of how dendritic and synaptic currents potentially shape neuronal output. The evidence supporting the tool's capabilities is **convincing**, with well-documented code, algorithmic innovation, and application to hippocampal pyramidal neurons. The work will be of interest to computational and systems neuroscientists seeking accessible methods to examine dendritic computations.

# Referee report: Reviewer #1 (Public review)

**DOI:** 10.7554/eLife.108352.3.sa1

Summary

Fogel & Ujfalussy report an extension of a visualization tool that was originally designed to enable an understanding of detailed biophysical neuron models. Named "extended currentscape", this new iteration enables visual assessment of individual currents across a neuron's spatially extended dendritic arbor with simultaneous readout of somatic currents and voltage. The overall aim was to permit a visually intuitive understanding for how a model neuron's inputs determine its output. This goal was worthwhile and the authors achieved it. Demonstrating the utility of extended currentscape, the authors leverage their models to generate interesting and detailed biophysical insights into widely studied neurophysiological phenomena with clear behavioral relevance. Overall, this study provides a valuable and well-characterized biophysical modeling resource to the neuroscience community.

Strengths

The authors significantly extended a previously published open-source biophysical modeling tool. Beyond providing important new capabilities, the potential impact of extended currentscape is boosted by its integration with preexisting resources in the field.

In keeping with the authors' goal to provide an approachable platform with intuitive visualizations of how current flows through neurons, the manuscript is approachable to non-computationalists. In particular, a dedicated glossary and elegant illustrations in Figure 2 boost accessibility for biologists.

Extended currentscape produces intriguing and detailed predictions spanning neurophysiological phenomena such as local dendritic spikes, complex spike generation, and feature selectivity (hippocampal place fields). By triggering analysis of modeled synaptic inputs on these events, the authors trace their origins from dendritic integration to synaptic input patterns.

The authors cleverly apply a graph theoretical approach to efficiently model bidirectional current flow throughout a neuron's dendritic arbor. As a result, extended currentscape can run on a standard personal computer.

The code is well-documented and freely available via GitHub.

Weaknesses

While extended currentscape meets its objective of modeling and illustrating the propagation of axial currents throughout a model neuron in great detail, it requires simulation and measurement of synaptic input currents. For this reason, there currently exists a very high technical barrier to conclusively test its intriguing predictions: simultaneous readout of synaptic inputs throughout a neuron's dendritic arbor. Mitigating this weakness, the authors propose a relatively more feasible alternative approach in Discussion: simultaneous voltage imaging of dendrites and their soma while estimating synaptic inputs from the distributions of voltage dynamics along individual dendritic branches.

# Referee report: Reviewer #2 (Public review)

**DOI:** 10.7554/eLife.108352.3.sa2

The electrical activity of neurons and neuronal circuits is dictated by the concerted activity of multiple ionic currents. Because directly investigating these currents experimentally is not possible with current methods, researchers rely on biophysical models to develop hypotheses and intuitions about their dynamics. Models of neural activity produce large amounts of data that are hard to visualize and interpret. The currentscape technique helps visualize the contributions of currents to membrane potential activity, but it is limited to model neurons without spatial properties. The extended currentscape technique overcomes this limitation by tracking the contributions of the different currents from distant locations. This extension allows tracking not only the types of currents that contribute to the activity in a given location, but also visualizing the spatial region where the currents originate. The procedure is first illustrated in a simple setting that allows testing its validity in an intuitive situation where a cell with an apical trunk and two dendritic branches responds to synaptic inputs. The procedure is then applied to study the initiation of complex spike bursts in a model hippocampal place cell.

The extended currentscape method represents a significant improvement over the original technique, which is already utilized by several research groups. By enabling the analysis of current contributions in spatially extended models, this technique provides a new lens for investigating neuronal and circuit dynamics and will be of use to the modeling community.

Comments on revisions:

The changes in Figure 2 greatly improved the manuscript.

# Author comment: Author response

**DOI:** 10.7554/eLife.108352.3.sa3

**Authors:** Bence Fogel, Balazs B Ujfalussy

**Affiliations:** HUN-REN Institute of Experimental Medicine Budapest Hungary; HUN-REN Institute of Experimental Medicine Budapest Hungary

The following is the authors’ response to the original reviews.

**Public Reviews:**

**Reviewer #1 (Public review):**

Summary:

Fogel & Ujfalussy report an extension of a visualization tool that was originally designed to enable an understanding of detailed biophysical neuron models. Named "extended currentscape", this new iteration enables visual assessment of individual currents across a neuron's spatially extended dendritic arbor with simultaneous readout of somatic currents and voltage. The overall aim was to permit a visually intuitive understanding for how a model neuron's inputs determine its output. This goal was worthwhile and the authors achieved it. Their manuscript makes two additional contributions of note: (1) a clever algorithmic approach to model the axial propagation of ionic currents (recursively traversing acyclic graph subsections) and (2) interesting, albeit not easily testable, insights into important neurophysiological phenomena such as complex spike generation and place field dynamics. Overall, this study provides a valuable and well-characterized biophysical modeling resource to the neuroscience community.

Strengths:

The authors significantly extended a previously published open-source biophysical modeling tool. Beyond providing important new capabilities, the potential impact of "extended currentscape" is boosted by its integration with preexisting resources in the field.

The code is well-documented and freely available via GitHub.

The author's clever portioning algorithm to relate dendritic/synaptic currents to somatic yielded multiple intriguing observations regarding when and why CA1 pyramidal neurons fire complex spikes versus single action potentials. This topic carries major implications for how the hippocampus represents and stores information about an animal's environment.

Weaknesses:

While extended currentscape is clearly a valuable contribution to the neuroscience community, this reviewer would argue that it is framed in a way that oversells its capabilities. The Abstract, Introduction, Results, and Methods all contain phrases implying that extended currentscape infers dendritic/synaptic currents contributing to somatic output., i.e. backwards inference of unknown inputs from a known output. This is not the case; inputs are simulated and then propagated through the model neuron using a clever partitioning algorithm that essentially traverses a biologically undirected graph structure by treating it like a time series of tiny directed graphs. This is an impressive solution, but it does not infer a neuron's input structure.

We are sorry if our text could be interpreted as if we were inferring unobserved inputs from the known outputs. This was not intentional and we were unaware of the possibility of such interpretation.

In fact, at the beginning of the Results, we started the description of the extended currentscape method by explicitly stating that we need to measure the input currents: “Our method … requires measuring the membrane and axial currents throughout the dendritic tree of a neuron (in every node of the circuit)”.

To further clarify that our method starts with measuring the input currents, we made this information explicit already in the abstract (“Our approach relies on the iterative decomposition of the axial current flowing between neighbouring compartments in proportion to the underlying membrane currents measured in the model.”), and in the Introduction (“Even if the membrane currents are known, studying the impact of particular ion channels on the neuronal response in such a dynamical system under *in vivo* conditions is hindered by two major obstacles”). We also rewrote several parts of the text to remove any phrases that could imply the inference of the inputs (line 568). We believe that after clarifying this at the beginning of the paper, the readers will not misinterpret our descriptions later in the text.

Because a directed acyclic graph architecture is shown in Figure 2, it is unintuitive that the authors can infer bidirectional current flow, e.g. Figure 3 showing current flowing from basal dendrites and axon to soma, and further towards the apical dendrites. This is explained in Methods, but difficult to parse from Results amidst lots of rather abstract jargon (target, reference, collision, compartment). Figure 2 would have presented an opportunity to clearly illustrate the author's portioning algorithm by (1) rooting it in the exact morphology of one of their multicompartmental model neurons and (2) illustrating that "target" and "reference" have arbitrary morphological meanings; they describe the direction of current flow which is reevaluated at each time step.

We thank for this comment. We agree that the concepts introduced here to explain our method are rather abstract and could be difficult to understand. To help the reader we followed the instructions of Reviewer and redesigned Fig. 2 to provide a step by step explanation of the extended currentscape method. In particular,

We used a simpler model where the structure of the graph can be directly related to the morphology of the model.

We show that the target node can connect multiple subtrees with axial currents flowing in different directions. We explain that in this case the inward and the outward subtrees are pruned and partitioned separately.

We provide a glossary in Table 1 to ensure that the readers can follow our description and do not get lost amidst lots of rather abstract jargon.

We also clarified that although the target compartment is chosen arbitrarily by the user, it remains the same for all time points throughout the analysis.

Analyses in Figure 7, C and D, are insightfully devised and illuminating. However, they could use some reconciliation with Figure 5 regarding initiation of individual APs versus CSBs within place fields.

We thank the reviewer for the positive comments and also for pointing out the potential source of misunderstanding. We slightly changed the text at Fig 5 to emphasize that this is a single example trial, and we added the following sentence to the paragraph describing Fig 7CD: “Consequently, the somatic current dynamics before the iAP and the CSB presented in Fig 5Cc-Dd can be regarded as illustrative samples from a broad distribution, but the differences observed between them are not representative.}”

The intriguing observations generated by extended currentscape also point to its main weakness, which the authors openly acknowledge: as of now, no experimental methods exist to conclusively tests its predictions.

We agree with the Reviewer that not being able to apply our extended currentscape method to reveal the current types driving real neurons recorded *in vivo* is currently a weakness of our approach. However, we would like to emphasize that it may be feasible to use it to estimate the spatial distribution of the membrane currents driving the cell based on *in vivo* voltage imaging data, as we briefly outline in the discussion.

**Reviewer #2 (Public review):**

Summary

The electrical activity of neurons and neuronal circuits is dictated by the concerted activity of multiple ionic currents. Because directly investigating these currents experimentally isn't possible with current methods, researchers rely on biophysical models to develop hypotheses and intuitions about their dynamics. Models of neural activity produce large amounts of data that is hard to visualize and interpret. The currentscape technique helps visualize the contributions of currents to membrane potential activity, but it's limited to model neurons without spatial properties. The extended currentscape technique overcomes this limitation by tracking the contributions of the different currents from distant locations. This extension allows tracking not only the types of currents that contribute to the activity in a given location, but also visualizing the spatial region where the currents originate. The method is applied to study the initiation of complex spike bursts in a model hippocampal place cell.

Strengths. >

The visualization method introduced in this work represents a significant improvement over the original currentscape technique. The extended currentscape method enables investigation of the contributions of currents in spatially extended models of neurons and circuits. >

Weaknesses.

The case study is interesting and highlights the usefulness of the visualization method. A simpler case study may have been sufficient to exemplify the method, while also allowing readers to compare the visualizations against their own intuitions of how currents should flow in a simpler setting. >

We thank the reviewer for this comment. In fact we had been also considering to include a simpler case study to illustrate the extended currentscape method in the original submission. In accordance with the comments from Reviewer 1, we now use a simple model to introduce the concepts in Figure 2 and provide a few examples where the reader can compare the results with their own intuition in simpler cases.

**Recommendations for the authors:**

**Reviewing Editor Comments:**

(1) Model complexity vs. intuition/validation. The case study relies on a very complex CA1 model, making it difficult to build intuition about current flow and to validate the visualization. Inclusion of a simpler benchmark (e.g., soma plus a dendrite with two branches, fewer compartments) is recommended to demonstrate how the extended currentscape behaves in a more tractable setting.

Inspired by the suggestions of the Reviewers, we modified Figure 2 and now first use a simple model with a soma and a dendrite with two branches to introduce the concepts of our analysis. We start with a few examples where the reader can compare the results with their own intuition in simpler cases.

(2) Rationale and citations for input structure. The in vivo-like input design (untuned inhibition; 12 co-tuned excitatory clusters with large conductances; the goal of generating place fields) would benefit from a more explicit rationale and substantially more literature support. Alternative plausible scenarios (e.g., distributed co-tuned inputs and homosynaptic plasticity) should be articulated, and choices situated within the experimental literature on CA1 excitation/inhibition, including tuning and anti-tuning results.

We extended the paragraph in the Results describing the input structure and added the most important references there. We added further references to the Methods section where we argue that “Reliable place cell tuning can be achieved by functional synaptic clustering without increased excitatory drive in the place field (Ujfalussy and Makara 2020) or via strong excitatory drive without input clustering (Grienberger et al., 2017, Ujfalussy and Makara, 2020). However, experimental data indicates that both of these mechanisms are present and contribute to the activity of place cells (Adoff et al., 2021,Tasciotti et al., 2025)” and “although interneurons can display spatial tuning, they typically have a broad tuning with low selectivity (Ego-Stengel et al., 2007, Dupret et al., 2013, Geiller et al., 2020). A weak disinhibition within the place field can also contribute to the selective firing of place cells (Geiller et al., 2022, Valero et al., 2022), this was not necessary for place cell activity in novel environments (Geiller et al., 2022) and the overall inhibitory input to place cells is largely untuned (Grienberger et al., 2017).”

(3) Scope of PCA-based claims. The interpretations derived from the PCA analysis appear broader than warranted, given subcellular heterogeneity and the dominance of somatic action potential variance. These claims should be tempered with more explicit statements about what PCA can and cannot resolve in this context.

We thank the Reviewer for the opportunity and encouragement to clarify this part of the text. We agree with the Editor and the Reviewers that the results of the PCA analysis can not be used to support claims regarding the presence or the absence of independent dendritic events. In fact, we aimed to use it as an illustration that global activity tends to dominate PCA analysis even when the “neuron is mainly driven by strong, functionally clustered synaptic inputs to a few dendritic branches”. We acknowledge that we did not formulate this point clearly in the original submission. Therefore we substantially rewrote this part of the Results and performed additional analysis to clarify that there is a substantial amount of soma-independent dendritic activity in our model that remains invisible for a PCA based analysis.

**Reviewer #1 (Recommendations for the authors):**

Major concerns:

(1) Depolarization-inactivated K+ may be an important consideration to model burst-firing.

Our current model includes 2 kinds of transient K+ channels that show inactivation after depolarization: a proximal and a distal type, as the original model in Jarsky et al., 2005. We now made this explicit in the main text (line 178).

(2) Description of the in vivo-like model's excitatory and inhibitory input structure needs many more citations of biological studies to communicate rationale for the author's decisions, e.g. untuned inhibitory neurons, organization of a subset of excitatory inputs into 12 function synaptic clusters with co-tuned presynaptic neurons and outsized synaptic conductances. The goal is clearly to create CA1 pyramidal neurons with place fields, which would be helpful to state upfront. But additionally, (a) place fields could arise from homosynaptic potentiation of distributed co-tuned excitatory inputs (e.g., Bittner, et al. 2017 study describing BTSP made no assumptions) and (b) CA1 inhibitory interneurons can be spatially tuned (Ego-Stengel & Wilson, 2006; Wilent & Nitz, 2007; Geiller, et al. 2020) and even anti-tuned (Geiller, et al. 2021).

We thank the Reviewer for pointing out the lack of appropriate references in this section. We made the following changes in the manuscript:

(1) Stated explicitly that the goal was to create place cell activity.

(2) Added references to the main text to justify our choices of the inputs (lines 234-241).

(3) We included a longer rationale for the choice of synaptic clusters and the lack of inhibitory (anti-)tuning in the Methods section, describing the neuron model. In brief, Adoff et al., 2021 reported more clustering of excitatory inputs within the place field. In our model, the degree of clustering is somewhat larger than the clusters reported. Although inhibitory neurons can be tuned, their tuning is much weaker than that of place cells and seems to play only a minor role in the generation of place fields (Grienberger et al., 2017). The presence of inhibitory anti-tuning is controversial: although Geiller et al., 2021 reported weak (~10%) anti-tuning, they did not find it in novel environments, indicating that it is not needed for spatially selective activity (lines 628-646).

(3) Interpretation of principal component-based analyses shown in Figure 4 could be toned down. As written in section "CSBs in the CA1 pyramidal neuron", it sounds like CA1 pyramidal neuron dendrites display minimal autonomous activity. However, PCA does not seem well-suited to address the heterogeneity of subcellular voltage dynamics over physiologically relevant timescales. Somatic action potentials, and their backpropagation/modulation of dendritic voltage, would of course explain a very large fraction of variance. However, if local dendritic events summate over fine timescales to initiate somatic firing, it is hard to imagine this important nuance being detected. On the other hand, it is hard to imagine single dendritic branches driving robust somatic firing except in the relatively extreme situation in which large numbers of synapses synchronously drive the same branch to initiate a local Ca2+ spike (Figure 3, A-C).

We agree with the reviewer that PCA can not reveal the potential dendritic origin of somatic APs, and thus is not suitable to assess the role of local dendritic spikes in shaping the output of the cell. We wanted to highlight here that even in cells with excitable dendrites driven by strong, local input clusters, exhibiting frequent local dendritic spikes, the dendritic membrane potential dynamics will be dominated by global fluctuations with surprisingly little sign of local dynamics in the PCA components. As the reviewer also pointed out, this may not be surprising as local events either remain spatially restricted and thus contribute little to the overall variability of the dendritic Vm or they initiate somatic APs and will thus be counted as global events.

To demonstrate the high propensity of local dendritic events, we analysed local Vm peaks in dendritic branches and found that ~7.6% of the peaks were not coupled to somatic APs.

Although this number could seem low, we emphasize that most of the 92.4% of the dendritic peaks coupled to APs potentially reflect the backpropagation of the same somatic events to multiple dendritic sites. To confirm this, we performed an additional analysis measuring the spatial extent (number of branches involved) of the individual dendritic events. We found that 90% of the events remained local, restricted to a few dendritic branches, while 10% of the events were global, associated with BAPs and involving the majority of the dendritic tree. Interestingly, these global events dominate the PCA analysis and are responsible for >90% of the dendritic Vm peaks. These results are included in a new panel in Figure 4H.

We conclude that, “this way, although only 10% of the dendritic Vm events were associated with bAPs, they were ~60-times larger than local events and they dominated the PCA analysis even in the presence of local regenerative dendritic events driven by strong, functionally clustered synaptic inputs.” We believe that this model and analysis could serve as an important benchmark for future experimental studies investigating the structure of membrane potential correlations in *in vivo* voltage imaging data (Lee et al., 2026).

(4) One suggestion would be to display more data as shown in Figure 4F, with a longer X axis to clarify the temporal relationship between local dendritic spikes and the first somatic action potential.

We added a few more examples including the CSBs presented in Fig8G-I as a new supplementary Figure S4. We also slightly extended the x-axis on this supplementary figure as the reviewer requested.

If the models indicate that passively filtered EPSPs drive most somatic action potentials, as seems to be the case in Figure 5, then this would also be helpful to show as in Figure 4F.

In Fig 5 we showed two examples of isolated APs. The first AP was indeed driven by passively filtered EPSPs. The second one was preceded and possibly caused by a dendritic spike, as highlighted by the black arrowhead labelled c in Fig. 5Cc. We further analysed the currents driving iAPs in Fig 7B and C, and found that there is considerable heterogeneity in the magnitude of the dendritic Na currents driving the soma before action potentials. Figure 8 and Figure S3 (now Fig. S5) show further examples for iAPs driven either by passively filtered EPSPs or dendritic spikes. We also included these examples in the new supplementary Figure S4.

(5) Another suggestion would be to use one-hot vectors containing onset times of different event types, since this would divorce the amplitude/duration of events from their influence over total variance.

In this paper our goal was to illustrate the ability of the extended currentscape method to reveal the origin of the axial currents driving neuronal activity. In Fig. 4, our primary intention was to characterize the membrane potential response of the model in a way that is easily comparable with experimental data. To further quantify the frequency of local events, we added a new panel showing the spatial extent of dendritic events (Fig. 4H). To make our model more comparable with recent publications, we also calculated two additional metrics used to evaluate the relationship between somatic and dendritic activity (Fig 4I-J). We hope that these additional analyses help the reader to characterize the prevalence and impact of local dendritic events on somatic activity.

(6) From section "Input conditions for complex spike burst generation", paragraph 2: "Note that synapse density, the ion channel mechanisms and the input statistics are identical for tuft and oblique branches,...". The authors should justify this parameterization given the numerous known differences between tuft and oblique branches in both of these regards and acknowledge accompanying interpretational caveats.

We agree with the reviewer that experimental data demonstrated several significant differences between the tuft and oblique branches regarding both the inputs they receive and the way they process it. However, in the present paper we chose not to include these differences for several reasons:

Here we aimed to focus on the abilities of the dendritic currentscape methods and use CSBs as a case study to illustrate how dendritic currentscape can reveal the membrane currents underlying complex neuronal responses.

Currently there is no CA1PN model that would be able to reproduce all data regarding tuft and oblique integration and would be able to fire calcium spikes. We only wanted to make minimal modifications to the existing CA1PN model to make it capable of generating Ca-spikes and CSBs. We are currently working towards developing and extensively testing a new model, examining the role of these regional differences in CSB generation.

Although there is information regarding input statistics and dendritic physiology in the literature, many of the relevant parameters are underconstrained. We wanted to avoid overfitting by keeping the model simple.

By maintaining identical inputs and ion channel distribution we can distinctly highlight the special role of tuft morphology in CSB generation. Altering the inputs or the ion channel density for the tuft would make the interpretation more ambiguous, and elucidating the specific role of the different factors in CSB generation is the subject of future investigations.

In sum, although we acknowledge that our model does not reflect the full complexity of CA1 PNs and its inputs, we regard this simplicity as a useful feature of the model. We added a section discussing potential future extensions of the model and highlighting interpretational caveats in the discussion (lines 482-490).

(7) Given the debate in the field regarding the level of functional autonomy present in dendrites, the authors' finding that dendritic voltage largely tracks that of the soma (though see concern above re: PCA), and their access to specific currents, the authors have an important opportunity investigate the divergence between Ca2+ and voltage sensors as reporters of dendritic activity.

For instance, why have some studies reported relatively common isolated dendritic Ca2+ transients in CA1 pyramidal neurons while other studies, including voltage imaging studies, have reported the opposite?

We thank the Reviewer for the opportunity to highlight a few important points regarding functional autonomy of dendrites based on the analysis of our model. We would like to first note that only parallel calcium and voltage imaging studies will be able to ultimately resolve this debate. Nevertheless, below we briefly summarize our take on this issue.

(1) In general, most Ca2+ imaging studies found that soma-independent dendritic events are rare. "Isolated dendritic transients (no coincident somatic event; see fig. S6, C and D, for example) were overall rare. Isolated apical dendritic Ca2+ transients, which have not previously been reported in CA1PNs, were larger and more frequent than those observed in basal dendrites." (O’Hare et al., 2022). "Activity in the ... basal dendrites ... along the track but outside of the place field was rarely observed” (Sheffield and Dombeck, 2014) and “overall, isolated dendritic transients were similar in size but occurred far less frequently than coincident dendrite-soma transients”, or “data indicate that spatially reliable dendritic firing was almost exclusively yoked to somatic tuning, likely reflecting strong backpropagation of burst firing during traversals of the somatic PF” (Rolotti et al., 2022). Consistent with this observation, a dendritic Vm peak chosen randomly from any branch has ~93% probability to be related to a bAP in our model. However, it is also true that ~90% of events in the model are local events, simply because isolated events involve ~60-times fewer branches (1.8 on average) than events associated with bAPs (114 branches) in the model. If the spatial extent of typical local events are also similarly small in real neurons as in the model, then even rare occurrences of dendritic events may reveal substantial dendritic independence. We added a section quantifying the functional autonomy of dendrites in the model in the main text, around Fig 4H.

(2) Ca2+ indicators are slower and nonlinear and thus they are somewhat unreliable reporters of dendritic voltage events, especially in distal dendrites (Wu et al., 2026; Gonzalez et al., 2026). To illustrate this, we calculated three metrics in our model that were also reported in recent dendritic Ca2+ imaging studies (Rolotti et al., 2022, Sheffield et al., 2014, 2017). First, we calculated the fraction of bAPs detected in a branch (called dendrite-soma coupling in Rolotti et al., 2022, see their Fig. 2C) as a function of the distance of the branch from the soma (our new Fig. 4I). In the Ca2+ imaging data, this was essentially constant ~30% between distances 5-100 µm from the soma. In contrast, the fraction of bAPs detected in the model was 100% in this range as bAPs propagation failures did not occur before µ100 µm. This is also consistent with a recent voltage imaging study showing that even low-transmission bAPs reliably propagate to the proximal dendrites (Lee et al., 2026, Fig 3G). The low and distance independent dendrite-soma coupling reported by Rolotti et al. can only be reconciled with the known biophysics of neurons if the recorded calcium signal is unreliable reporter of the underlying voltage. Indeed, it has been reported that Ca signals associated with bAPs can be absent in some dendritic branches (Landau et al., 2022) or that local, nonlinear Ca signals can appear in the absence of local regenerative voltage response (Weber et al., 2016, Tran-Van-Minh et al., 2016) and that the Ca signals are highly variable across cells (Eltes et al., 2019).

Second, we calculated the fraction of local events as a function of the distance from the soma (our Fig 4J; see also Fig. 2F in Rolotti et al.). When averaged across all branches, this was somewhat lower in the model (18%) than in the data (38%) which, again, could be explained by the low reliability of detecting global voltage events in all compartments based on the calcium signal.

Third, the range of branch-spike-prevalence (BSP) values in our model (0.5-0.9; Fig. 4H) seem consistent with that reported (0.4-0.8) at first (Fig 4C of Sheffield et al., 2014; Fig 2 of Sheffield et al., 2017). However, we note that there are several important differences: for technical reasons, Sheffield et al. reported BSP for place field traversals and not for individual events, and they measured Ca2+ dynamics in the basal dendrites. Since bAPs are almost always present in all basal dendrites in the model (basal BSP > 0.9 for all events with somatic spikes) and place field traversals were always accompanied by somatic APs, BSP for basal dendrites would be nearly 1 in the model. Thus, the lower BSP values reported by Sheffield et al. could be explained by the limited reliability of the Ca2+ indicators in reporting regenerative voltage events in neuronal processes.

We briefly discussed these differences in the Discussion (lines 474-478).

(3) Finally, to our knowledge, there are 3 relevant *in vivo* voltage imaging studies in CA1 PNs. Liao et al., 2024 found that in induced place cells the tuning of dendritic events (presumably local or back-propagating Na-spike) was similar to the somatic tuning, which is consistent with our model where dendritic activity and tuning is dominated by bAPs. However, they did not acquire simultaneous signals from the dendrites and the soma so they could not study the independence of the dendritic events. Lee et al. (2026) found that only 10% of the dendritic events are not associated with a somatic spike, which is lower than the number of independent events in the model. However, the events they found were generated in the distal apical trunk (their Fig 3D) and they could not record from the most distal branches where most of the isolated events were generated in our model. Gonzalez et al., 2026 measured voltage and calcium in selected locations within the dendritic tree, and could not reliably estimate the fraction of isolated events throughout the cell. (Gonzalez et al, 2024 measured voltage only in single spines and soma, but did not quantify independent dendritic events; Wong-Campos et al., 2023 measured dendritic integration and bAPs in L23 branches; Wu et al. 2026 recorded in CA2 neurons.)

We added a paragraph in the discussion comparing the level of functional autonomy present in the model dendrites to recent Ca- and voltage-imaging studies (lines 467-474).

Minor concerns:

(1) Abstract:

There is a need to explain what currentscape is - even at the cost of not invoking its name. To a reader not familiar with currentscape, the abstract is extremely difficult to understand.

We reworded the title and the abstract to make them more accessible to readers not familiar with the term currentscape.

(2) "Currentscape analysis of place field dynamics" section:

It would be helpful to emphasize upfront that dendritic determinants of individual somatic APs versus CSBs will be discussed separately. Since somatic action potentials are discussed before CSBs, I found this section initially confusing as I attributed those findings to CSBs until reading the next paragraph.

We added a sentence to clarify that we analysed subthreshold responses, APs and CSBs separately.

(3) Bottom of p2 discussing mixed literature on what drives CSBs in CA1 PCs:

Overall accurate and useful point, but an important nuance is glossed over which misportrays state of field. References ex vivo studies that fail to drive CSBs with somatic current injection and in vivo study successfully doing so. These aren't really conflicting results. In vivo current injection co-occurs with spontaneous synaptic input, which is high in CA1 and results in PCs that are significantly depolarized at rest relative to those in acute slices. Bittner 2017 ex vivo results are consistent with this: CSBs driven by Cs+-based internal solution to block K+ channels (partially, using strategy of purposefully high series resistance). Similar situation in vivo given that A-type K+ channels are inactivated by depol. Resulting increase in input resistance lowers input threshold to CSB. This is clarified in Results, p.5: "Under in vivo-like synaptic input conditions (see below and Methods), dendritic Ca2+-spikes could also be evoked by somatic current injection (Fig. S1E), as in Bittner et al. (2015).", which makes p. 2 feel especially awkward.

We agree with the Reviewer that these are not necessarily conflicting results. We rephrased this section, emphasizing that the role of the different input pathways in the initiation of CSBs are not clear.

(4) Abbreviating "pyramidal neuron" with PC is confusing:

PC often means place cell. The authors could change this, such that PC refers to "pyramidal cell", or else use PN as an abbreviation. It is important to avoid confusion, especially because place cell dynamics feature prominently in the manuscript.

Thanks for the suggestion. We replaced PC with PN throughout the manuscript.

(5) Only apical dendritic parameters are described in section 2 of Results, but the full morphology is shown in Figure 3B with basal currents shown in panels C and F. Some clarification is needed - either what currents were considered for basal dendrites and why, or else why basal dendritic current parameters were not considered for this simulation using apical dendritic current injection but nonetheless examining basal dendritic currents.

We clarified in the text that the original model contained a standard set of Na and K channels (line 178).

(6) Clarify "i" and "s" in the Figure 3C legend - "intrinsic" and "synaptic" white letterings are small/hard to see in the bottom subpanels.

We now spell out intrinsic and synaptic in the Figure and increased the contrast of the letterings.

(7) Regarding the computational benefit of recursively decomposing axial currents along an adaptively truncated acyclic graph, it would be useful to (a) include a supplemental figure benchmarking this approach to standard approaches to quantify the described gain in computational efficiency and (b) describe computing hardware in the Methods.

We included an estimated benefit of the pruning process (line 758) as well as the utilised computing hardware and the simulation times in the Methods (line 776).

**Reviewer #2 (Recommendations for the authors):**

The manuscript is in great shape, it is well organized, and the figures are gorgeous. I believe that the extended currentscape is a great extension of the original currentscape method. In particular, the possibility of partitioning currents by the spatial location of their sources is a great addition. >

Recommendations:

(1) The method is applied in the context of an interesting case study that highlights its usefulness. However, the model in the study is so complex that it is difficult to develop an intuition of how currents should be flowing, and this makes it hard to intuitively validate the visualization method. I think that applying the extended currentscape in a simpler model - maybe a soma with a dendrite with two branches, fewer compartments - would be instrumental in developing this intuition. >

We now first use a simple model with a soma and a dendrite with two branches to introduce the concepts in Figure 2 and provide a few examples where the reader can compare the results with their own intuition in simpler cases. We also added the currentscape analysis of a standard, two-compartmental model from Pinsky and Rinzel, 1994 as Supplementary Figure 1.

(2) I found a number of typos and minor stylistic details you may want to fix in a revised version of the manuscript.

(a) Abstractine, line 12. I believe the word "recursive" is a bit technical at this point. It's meaning in this context becomes clear after ones goes through the details of the algorithm (Figure 2). >

We replaced the word “recursive” with “iterative”. We hope that this will make the abstract clearer for the readers. In fact, we realized that the word iterative is a better description of the algorithm, so we replaced the “recursive” with “iterative” consistently throughout the manuscript.

(b) Figure 1, caption."Since we included the capacitive current, the magnitude of the inward and the outward currents is identical (Kirchhoff's law)."This sentence can be confusing. If the inward and outward currents are the same, the membrane potential doesn't change. I believe that you are including the capacitive current in the inward (or outward) currents.

Indeed, we included the capacitive current in the inward or outward currents. We changed the text to clarify this.

(c) Lines 92-93. I do not fully understand this sentence. Are you making an assumption? What does 'continuos flow of axial current' mean? >

By ‘continuous flow of axial current’ we meant a spatially continuous stream of axial currents flowing from the reference to the target. To clarify this, we added the explanatory sentence: “i.e., if the axial current is not blocked or reversed between the reference and the target.”

(d) Equation (1.) Why summing axial currents over j? Is this for the case of a branching point?

The compartment could be (1) part of a continuous segment of dendritic branch, where axial currents can flow from the distal and the proximal direction (sum over 2); (2) It can be a branch point with 3 axial currents; (3) or it can be a leaf compartment with only one axial current, in which case the summation is not relevant. We clarified this in the text.

(e) Figure 2, caption. Typo. "When the axial currents flows…" Should it be 'current'? - Figure 3, caption. Typo in (C) "Extended currentscape" >

Corrected.

(f) Figure 4. I cannot see the grey lines or the dotted lines mentioned in the caption. >

We added an arrow highlighting the gray and the dotted lines in the figure.

(g) Figure 5, caption. "Red boxes highlight regions analyzed in panels B-D."Because this is a spatially extended model, region may be confused with spatial location, but you are highlighting a temporal interval. >

We rephrased the caption referring to temporal intervals now.

(h) Line 341. This is a numerical experiment, correct? >

We clarified in the text and added that it was indeed a simulation experiment.

(i) Line 349. Should it be 'distributions'? >

Corrected

(j) Line 422. Typo. Missing space 'in vivousing' >

Corrected

(k) Line 537. "Preprocessing membrane…" I found this entire subsection a bit confusing and hard to read.

We rephrased this subsection to clarify it and facilitate reading.
